# The prevalence and correlates of common mental disorders among prisoners in Addis Ababa: an institution based cross-sectional study

**DOI:** 10.1186/s13104-019-4425-7

**Published:** 2019-07-12

**Authors:** Alemseged Solomon, Getnet Mihretie, Getachew Tesfaw

**Affiliations:** 1 Department of Non-communicable Diseases, Addis Ababa Correctional Institution, Addis Ababa, Ethiopia; 2 Department of Psychiatry, College of Medicine and Health Sciences, University of Gondar, Gondar, Ethiopia

**Keywords:** Common mental disorder, Prisoner, Correctional Institution

## Abstract

**Objective:**

About one in seven prisoners is diagnosed with common mental disorders whose global prevalence ranges from 13 to 92.5%. The problem negatively affects the physical, psychological, and social well-being of prisoners. However, research into common mental disorders and associated factors among prisoners in low and middle-income countries has been limited. Therefore, this study aimed to explore the common mental disorders and associated factors among prisoners in Ethiopia to contribute the attempt to ensure optimal care for prisoners.

**Results:**

The prevalence of common mental disorders among prisoners was found to be 58.4% [95% CI 53.70, 63.00]. In the multivariable logistic regression, poor social support [AOR = 2.4, 95% CI 1.16, 4.85], economic crisis [AOR = 3, 95% CI 1.84, 4.85], secondary school education [AOR = 2.3, 95% CI 1.04, 5.20], unemployment before arrest [AOR = 1.7, 95% CI 1.04, 2.80], and history of psychiatric illness [AOR = 4.3, 95% CI 1.21, 15.56] were factors significantly associated with the problem.

**Electronic supplementary material:**

The online version of this article (10.1186/s13104-019-4425-7) contains supplementary material, which is available to authorized users.

## Introduction

Common mental disorder (CMD) is a term used to describe a group of mental disorders that frequently include depression, anxiety, and somatoform which are highly prevalent in low and middle-income countries [1–3]. According to estimation, over 450 million people in the world suffer from some form of mental disorder and one in four whom met the criteria in their lives have had mental disorders [1, 4].

The prevalence of the CMD which is 14% today, is predicted will rise to 15% by 2020, becoming the second leading cause of ill health in low-income countries [5, 6]. Over 10.2 million people held in penal institutions with restricted freedom, autonomy, and communication with their families have their physical, psychological, and social well-being affected throughout the world [6, 7]. The magnitude of CMD is higher among prisoners than in the general public [8–10]. Common mental disorders which may be present before arrest might be further aggravated after imprisonment [3, 6].

Many prisoners, at least one CMD and nearly one in seven of those have such history, suffering from CMDs [11, 12]. The overall magnitude, of CMD among prisoners ranges from 13 to 92.5% [11, 13]. In addition to imprisonment, the variety of other factors, like the prison environment, educational status, overcrowding, crime type, term of sentence and isolation from social networks have roles in the development of CMDs [1, 6, 8, 14].

Evidence suggests that the presence of mental illness among prisoners contributes to an increased in the risk of suicide, violence, morbidity, and mortality [1, 10, 12]. Prisoners who have psychiatric disorders need to have access to mental health treatment and support at all stages of criminal justice [15]. Timely identification, treatment, and rehabilitation are almost missing in many prisons, particularly in developing countries [6]. However, research into common mental disorders and associated factors among prisoners in low and middle-income countries is limited. Therefore, this study aimed to assess the prevalence and associated factors of common mental disorders among prisoners in the correctional institution in Ethiopia with a view of informing the development of interventions.

## Main text

### Methods

#### Study setting and design

An institution-based cross-sectional study was conducted at Addis Ababa correctional centers from May to June 2015, Addis Ababa, Ethiopia.

#### Study population

All sampled prisoners with jailed at Addis Ababa correctional centers were excluded. Prisoners critically ill were excluded.

#### Sampling procedures

The sample size was determined by using the single population proportion formula with the assumptions of 35.5% prevalence of CMD from a study conducted in Southwest Ethiopia, Jimma [16], with 95% CI, and a 4% margin of error. Assuming a 10% non-response rate, 452 prisoners were recruited randomly by using the systematic sampling technique. The sampling interval was determined by dividing the total study population by the total sample size; then, the starting point was randomly selected.

#### Data collection

Data were collected using a pre-tested, interviewer-administered questionnaire which contained CMD, socio-demographic characteristics, clinical, and prison-related factors. We used a face to face interviewer administered questionnaire to collect data. Common mental disorders were measured by using Self Reporting Questionnaires-20 (SRQ-20), developed by the World Health Organization to screen CMD in primary health care settings in low-income countries [17]. However, owing to high illiteracy rate in Ethiopia and other countries of the same status, the tool was used in an interview format, validated and subsequently used for epidemiological, clinical and community settings in the country. The scales used as cutoff scores for CMD were ≥ 8 [17].

Social support was assessed by the Oslo 3-item social support scale which had a 3-item questionnaire commonly used to assess social support and used in several studies. The sum score scale ranges from 3 to 14 with three broad categories: “poor support” 3–8, “moderate support” 9–11, and “strong support” 12–14 [18].

Data were entered into Epi-info 7 software after checking for completeness and transferred to the statistical package for social sciences version 20 for analysis. Bi-variable and multivariable logistic regression analyses were done to see the association of each independent variable with the outcome variable. The strength of the association was evaluated using the adjusted odds ratio with a 95% CI, and less than 0.05 P-values were considered statistically significant.

### Results

#### Socio-demographic characteristics

A total of 452 respondents took part with a response rate off 98.9%. The mean age of the respondents was 26 (± 10) years; almost half (50.1%) were female, 171 (38.3%) were single, and 303 (67.8%) were between the ages of 18 and 27 years (Table 1).

**Table 1:** Distribution of prisoners by socio-demographic characteristics at Addis Ababa correctional center, Addis Ababa, Ethiopia, in 2015 (n = 447)

Variables	Categories	Frequency	Percent
Sex	Male	223	49.9
	Female	224	50.1
Age in years	18–27	303	67.8
	28–37	83	18.6
	38–47	40	8.9
	48–57	15	3.3
	≥ 58	6	1.5
Marital status	Single	301	67.3
	Married	98	21.9
	Others^a^	48	10.8
Religions	Orthodox	334	74.7
	Muslim	56	12.5
	Protestant	49	11.0
	Others^b^	8	1.8
Ethnicity	Amhara	206	46.1
	Oromo	95	21.1
	Tigray	84	18.9
	Others^c^	64	13.9

^a^Divorce, widowed

^b^Jobba, Catholic

^c^Gurage, afar, and Somali

#### Psychosocial characteristics

Of the participants, 48.8% had poor social support; only 65 (14.5%) had chronic physical illnesses; 29 (6.5%) were diagnosed with psychiatric illnesses, and 23 (5.1%) were convicted once (Table 2).

**Table 2:** Distribution of psychosocial factors of prisoners at Addis Ababa correction center, Addis Ababa, Ethiopia, in 2015 (n = 447)

Variables	Categories	Frequency	Percent
Social support	Poor	218	48.8
	Moderate	162	36.2
	Strong	67	15.0
Having a chronic physical disease	Yes	65	14.5
	No	382	85.5
Seriously illness, injury or assault of a prisoner in last 6 months	Yes	93	21.0
	No	353	79.0
Death of relative in the last 6 months	Yes	37	8.3
	No	410	91.7
Break up of friendship or relationship in the last 6 months	Yes	77	17.2
	No	370	82.8
The financial crisis in the last 6 months	Yes	268	60.0
	No	179	40.0
Ever diagnosis psychiatric illness	Yes	29	6.5
	No	418	93.5
A family history of mental illness	Yes	38	8.5
	No	409	91.5
Problem with police/court in the last 6 months	Yes	73	16.3
	No	374	83.7
No job before arrested	Yes	204	45.6
	No	243	54.4
Suffered from violence in the last 6 months	Yes	63	14.1
	No	384	85.9
Ever convicted	Yes	29	6.5
	No	418	93.5
How often do you convict	Once	23	5.1
	Twice	2	0.4
	≥ 3 times	3	0.7

#### Prisoner characteristics

More than half (55%) of the study participants were accused of shoplifting, and for 233 (52.1%) the length of sentences were from 1 to 3 years duration. Over two in five (43.2%) have stayed in prison for 1 to 3 years (Additional file 1).

#### Prevalence of CMD among prisoners

This study revealed that the prevalence of CMD among the study participants was found to be 58.4% with 95% CI (53.7, 63.0) (Additional file 2).

#### Factors associated with CMD

Among all explanatory variables, poor social support, educational status, history of psychiatric illness, and economic crisis had less than 0.2 *P* value in the bi-variable logistic regression considered as the multivariate logistic regression model.

The multivariate analysis suggested that poor social support was about 2.4 times more risky for CMD compared to patients with good social support (95% CI 1.16, 4.85). Economic crises in the last 6 months was 3.95 times (95% CI 1.84, 4.85) more likely to lead to CMDs compared to its absence.

Similarly, secondary school education was two times (95% CI 1.04, 5.20) more likely to lead to CMD compared to tertiary level of graduates, and the odds of CMD increased by 1.7 times (95% CI 1.04, 2.80) for participants who had no jobs before arrest compared their counterparts. The odds of developing CMD were 4.3 times (95% CI 1.21, 15.56) higher among participants who had history of mental illness compared with those who had such no history (Table 3).

**Table 3:** Factors associated with common mental disorders among prisoners in Addis Ababa correctional center, Addis Ababa, Ethiopia, in 2015. (n = 447)

Variables	Categories	Common mental disorder		COR, 95% (CI)	AOR, 95% (CI)
		Yes	No		
Level of educational	Illiterate	37	17	2.96 (1.37, 6.41)	2.34 (0.87–6.32)
	Primary	116	89	1.78 (0.99, 3.18)	1.5 (0.70–3.38)
	Secondary	83	46	2.45 (1.31, 4.61)	2.3 (1.04, 5.20)*
	Above tertiary	25	34	1:00	1:00
Social support	Poor	147	71	3.1 (1.74, 5.40)	2.4 (1.16, 4.85)**
	Moderate	87	75	1.72 (0.96, 3.10)	1.8 (0.80–3.17)
	Strong	27	40	1:00	1:00
Financial crisis	No	67	112	1:00	1:00
	Yes	194	74	4.4 (3.00, 6.57)	3 (1.84, 4.85)***
Job before arrested	Yes	117	126	1:00	1:00
	No	144	60	2.6 (1.75, 3.83)	1.7 (1.04, 2.80)*
Ever diagnosed psychiatric illness	No	236	182	1:00	1:00
	Yes	25	4	(1.64, 14.10)	4.3 (1.21, 15.56)*

Significant association (* P-value < 0.05, ** P-value < 0.01 and *** P-value < 0.001), n = sample size P-value for Hosmer and Lemeshew test = 0.56 common mental disorder

### Discussion

In the current study, the prevalence of common mental disorders and its possible association with various factors was assessed. The results showed that a remarkable proportion of prisoners had common mental disorders. The prevalence of CMDs among prisoners was found to be 58.4%. Regarding prevalence, our result is consistent with those of other studies carried out in South Africa, Nigeria, Zambia, Brazil, and Iran where it was estimated at 55.4%, 57%, 63.1%, 56.1%, and 57%, respectively [9, 19–22].

On the other hand, our finding far higher than those of studies did in two areas of Ethiopia, Kenya, and Iran and reported 35.5, 50, 28.9, and 43.4%, respectively [8, 14, 16, 23]. The variation might be due to distinctions in the sample sizes, measurement tools, and the socio-culture distinctions between Ethiopia and the other counties.

In Dilla (south Ethiopia), Jimma (southwest Ethiopia), and Iran, a clinical outcomes, and the symptoms check list-90-revised questionnaire were utilized to screen and assess CMDs among prisoners, respectively [8, 14, 16]. On top of that, crime type and length of sentences could be considered as sources of variations.

The present study finding was lower than those of studies done in Egypt, the United States, South Wales, Australia, Iran, and Pakistan where the magnitude was estimated at 81.5%, 70%, 74%, 80%, 88%, and 85%, respectively [12, 20, 24–27]. The discrepancy might be due to measurement tools and sample sizes. In Egypt, 88 prisoners were included in the study to assess depression and anxiety disorders [12]. In Australian, 916 prisoners were studied using the Composite International Diagnostic Interview (CIDI) to test psychiatric illnesses [25], but in the US taking the global severity index of the brief symptom inventory was employed to assess psychiatric disorders [21]. More than twice as many participants of the current study were enrolled in South Wales to assess common mental illnesses by using CIDI among prisoners [20]. In Iran, the fourth edition of the diagnostic and statistical manual criteria was put to use to assess a lifetime diagnosis of psychiatric disorders among prisoners [22], and in Pakistan the beck depression inventory scale was used to test depression in people [24].

Regarding factors, CMDs were 2.3 times higher among respondents who had secondary school education compared to prisoners who had above tertiary level education. This finding supported by that of study done in the southern parts of Ethiopia [14].

Poor social support was 2.4 times more likely to develop CMD compared with good social support. The reason might be that poor social support is one cause of mental illness which affects the treatment outcomes of the problem.

Economic crises in the last 6 months were three times more likely to be a risk factor for CMD compared to situations with such crises. The reason might be that serious economic crises and financial problems are major socioeconomic risk factors for mental health disorders. Such crisis among prisoners contributes to declining quality of life, depression, suicide, and homicide.

Prisoners who were not employed before arrest were 1.7 times more likely to develop CMD than prisoners who had jobs. Unemployment is one of the factors for mental illnesses lead prisoners to CMD and repeated arrests. This finding is consistent with that of a study done in Nigeria [22].

Finally, history of psychiatric illness was 4.3 times more likely to cause CMD compared with such no history. This result is consistent with that of a study done in Egypt [12]. The reason might be that just like antisocial personality disorder, substance use disorder, and history of mental illnesses are strongly associated with criminals in that such history may aggravate CMDs during imprisonment.

### Conclusion

In study, the overall magnitude of common mental disorder was 58.4%. Poor social support, economic crisis in the last 6 months, history of mental illness, secondary level education, and unemployment before arrest were significantly associated with common mental disorders.

## Limitations

A cross-sectional study design, we cannot permit conclusions about some variables, for example, about deciding whether common mental disorder symptoms are at risk or a consequence. This finding of this work is likely only to hint at the complex interactions between CMDs and explanatory variables. Further research should be conducted on risk factors for CMDs to strengthen and broaden our results.

## Additional files

**Additional file 1.** Distribution of criminal and prison-related characteristics of prisoners in Addis Ababa correctional center, Addis Ababa, Ethiopia, in 2015. (n = 447).

**Additional file 2.** Prevalence of common mental disorders among prisoners in Addis Ababa correctional center, Addis Ababa, Ethiopia, in 2015. (n = 447).

## Data Availability

The dataset during and/or analyzed during the current study available from the corresponding author on reasonable request.

## Abbreviations

AOR: adjusted odds ratio
CMD: common mental disorders
CIDI: composite international diagnostic interview
OR: odds ratio
SRQ: Self Reporting Questionnaire
WHO: World Health Organization
UOG: University of Gondar
