# Supplementary material for: The prevalence and correlates of common mental disorders among prisoners in Addis Ababa: an institution based cross-sectional study
Source: BMC Res Notes. 2019 Jul 12;12:394. doi: 10.1186/s13104-019-4425-7 (PMC6624986; doi:10.1186/s13104-019-4425-7)
Supplement: Supplementary file 1 — Additional file 1. Distribution of criminal and prison-related characteristics of prisoners in Addis Ababa correctional center, Addis Ababa, Ethiopia, in 2015. (n = 447). [file 13104_2019_4425_MOESM1_ESM.docx]

Additional file1**.** Distribution of criminal and prison-related characteristics of prisoners in Addis Ababa correctional center, Addis Ababa, Ethiopia, in 2015. (n=447)

| Variables | Categories | Frequency | Percent |
| --- | --- | --- | --- |
| Type of crime | Theft | 246 | 55.0 |
|  | Fight | 128 | 28.6 |
|  | Murder | 29 | 6.5 |
|  | Cheating | 22 | 4.9 |
|  | Gangster | 17 | 3.8 |
|  | Drug trafficking | 5 | 1.1 |
| Length of sentence | <1 year | 51 | 11.4 |
|  | 1-3 years | 233 | 52.1 |
|  | ≥4 years | 163 | 36.5 |
| Length of stay in prison | <1 year | 76 | 17 |
|  | 1-3 years | 193 | 43.2 |
|  | ≥4 years | 178 | 39.8 |
