# Supplementary material for: The prevalence and correlates of common mental disorders among prisoners in Addis Ababa: an institution based cross-sectional study
Source: BMC Res Notes. 2019 Jul 12;12:394. doi: 10.1186/s13104-019-4425-7 (PMC6624986; doi:10.1186/s13104-019-4425-7)
Supplement: Supplementary file 2 — Additional file 2. Prevalence of common mental disorders among prisoners in Addis Ababa correctional center, Addis Ababa, Ethiopia, in 2015. (n = 447). [file 13104_2019_4425_MOESM2_ESM.docx]

Additional file 2. Prevalence of common mental disorders among prisoners in Addis Ababa correctional center, Addis Ababa, Ethiopia, in 2015. (n=447)

| Variables | Categories | Common mental illness | |
| --- | --- | --- | --- |
|  |  | Yes (%) | No (%) |
| Sex | Male | 130(29.1%) | 93(20.8%) |
|  | Female | 131(29.3%) | 93(20.8) |
| Educational level | Illiterate | 37(8.3%) | 17(3.8%) |
|  | Primary | 116 (26%) | 89(20%) |
|  | Secondary | 83(18.6%) | 46(10.3%) |
|  | Tertiary and above | 25(5.6%) | 34(7.6) |
| Employment | Yes | 60(13.4%) | 144(32.2%) |
|  | No | 117(26.2%) | 126(28.2%) |
| Social support | Poor | 147(33%) | 71(16%) |
|  | Medium | 87(19%) | 75(17%) |
|  | Strong | 27(6%) | 40(9%) |
| Ever diagnosis psychiatric illness | Yes | 25 (5.6%) | 4(1%) |
|  | No | 181(40.4%) | 237(53%) |
| Financial crisis | Yes | 194(43.4%) | 74(16.6%) |
|  | No | 67(15%) | 112(25%) |
